# American Gynæcological Association

**Published:** 1880-09

**Authors:** 


### AMERICAN GYNÆCOLOGICAL ASSOCIATION.

The following extract is taken from the Cincinnati Lancet and Clinic:

The fifth annual session of this association met in this city on September 1st, 1880, and was called to order by the president, Dr. Marion Sims.

After call of the roll by Dr. Chadwick, the president introduced Dr. Thad A. Reamy, of Cincinnati, who delivered the address of welcome.

"*What is the Proper Field for Battey's Operation*," was the title of the paper announced to be read by Dr. Robert Battey, of Rome, Ga.

Dr. Battey asked to be excused from presenting his paper in full as he had not been able to complete it. He would, however, lay before the society a brief outline of the points he intended to make in his paper. He said: "When I announced this operation to the profession in 1872, I foresaw that the field of its application must be very restricted; for the honor of the profession and in the interest of humanity I had fully expected the field to be more restricted than it has been. There are some things connected with its application from which all the instincts of a manly nature recoil with the utmost repugnance. It was my expectation that when the profession would concede me ground, although I felt absolutely sure it would with a certain restricted ground, it would be conceded to me grudgingly. I took the position from the start that this never would be an operation of election. It is not a question whether you shall submit a female to an extirpation of the ovaries or undertake some other operation. A question of election between this and any other resource of gynecology can never arise. Nothing could have induced me to go into the community I live in to extirpate the ováries of a female, to hasten the change of life, except a most solemn sense of duty. I think to day that no physician ought to sit down quietly and calmly and select this operation in preference to any other expedient that offers itself. The case ought to be narrowed down to this expedient or none at all. I stand upon that ground exactly to-day where I did in 1872. It is never desirable; but when necessary it becomes a stern duty from which a surgeon, having upon his hands the life and happiness of a human being, and in whose case he can only select between this expedient and worse and far more direful consequences, can not shrink.

I announced from the very start that the operation was applicable only to certain classes of cases. In the first place, they must be cases that are incurable by any other means; in the second place they must be cases menacing life, such as would allow him a policy of inaction; in the third place, they must be cases from which he may reasonably expect to relieve the patient of the direful consequences of their disease by a change of life. He must ask himself: If she had her change of life next week would she probably get well? If she would, I propose to substitute for the natural change of life an artificial one, and secure the same result artificially that nature accomplishes in the change of life. It has been my habit in all my cases to ask myself three questions: Is it a mortal case? Is it incurable by other known resources of the art? Is it curable by a change of life? If all these questions be properly answered, then the operation is a proper one. I foresaw at the outset that the conditions which must necessarily call for this operation cover a large part of the whole field of gynæcological practice. They must be very variable under different circumstances.

I noticed in my paper, first, a striking class of cases in which there is absence of the uterus with more or less irregular ovulation and a violent nervousness of the system. There is produced in the system irregularity of circulation, local congestion and a certain exalted nervous condition of the patient and producing direful consequences resulting from ovariation unrelieved by nature's assistance in the menstrual discharge. Such cases in which these violent perturbations are produced are absolutely incurable by any other resource of our art. There is no means of supplying a uterus to give rise to the monopoly supply of blood, and the only resource is to go to the other end of the case and extirpate the ovaries which are simply surplusage in the system. With the uterus there disappears these violent perturbations of the nervous system.

The second class of cases are those of which I reported an instance eight years ago. It was a case I reported on in New Orleans. The lady had been confined during the war without adequate medical attention. There was complete occlusion of the whole metro-vaginal canal. The case was operated on afterwards, with the hope of letting out the returning menses, but without success. I thought there was abundant reason to be satisfied there was no liquid substance in the uterus whatever. Without noting a vestige of the vaginal canal, my judgment was that the only possible remedy in the case was extirpation of the ovaries. She had these violent nervous and vascular perturbances. The result of the operation was highly gratifying.

There is another class of cases of which I have seen several instances. They are cases that I call menstrual mania or ovarian mania. The reason becomes dethroned by reason of violent perturbations attendant upon this stoppage of the menstruation. Sometimes it occurs even where the menses are present but its functions are deranged, where menstruation goes on with a certain degree of regularity but accompanied with the greatest degree of pain. With violent perturbations of the system these cases to which I allude, are, I believe, absolutely incurable by any other resource of the art. A careful investigation of the ovaries after they are removed shows why they are incurable. Because there are profound changes in the structure of the ovaries which it is impossible to expect any medicine to control. We might as well try to cure genuine tuberculosis. It is idle to talk of Virginia Springs curing this case; it is idle to talk of medicine curing it; it is idle to talk of any medical operation except their removal. When it becomes necessary to incarcerate here in an insane asylum, I cannot conceive of any case that appeals more strongly to our sensibilities and induces us to sacrifice the pernicious organs that are so destroying to the mind and body of the patient.

There is still another class of cases—a rather numerous class—those of ovarian epilepsy. Here, let me remark, that a distinction should be drawn between cases of clearly marked uterine epilepsy and ordinary epilepsy. I foresaw from the first, with great alarm and apprehension, that the announcement of this operation was likely to be followed by great abuse of it. I trembled then with regard to the consequences, and I tremble to-day in regard to the consequences that grow out of the misapprehension. I have attempted from the first to sound the alarm to the medical profession all over the world, to encourage them to be extremely cautious and circumspect in selecting cases for this operation. I am able to say to you that whatever may be the influence of that medical conservatism, I am unable to look over my own fifteen cases and single out in my calm, mature judgment to-day a single one in whom I feel I have done my patient the slightest harm. There is not one of the fifteen—including two fatal cases—that I would not operate on again to-day with the same facts and circumstances before me that I had at that time. It is, therefore, very necessary not to assume that each case of epilepsy that comes into our hands is a case of uterine epilepsy. It should appear affirmatively.

Another class is—and in announcing it I am required to admit that it is an entirely unscientific and inexact classification—what I would call amenorrhœa; there is absence of the menses; there is a pernicious amenorrhœa that is utterly destroying the life of the patient. And a few of these cases I have found it impossible to class under any other head than amenorrhœa. This condition of the patient, whatever the cause of it may be, justifies the removal of the ovaries. It cures the patient, and nothing else will. Of course it would be very wrong to state that under any circumstances this operation is a cure for amenorrhœa; but what I do say is, that there are some aggravated cases of amenorrhœa that are incurable by any other resource, and are curable by the removal of the ovaries.

There is another class of patients operated on by Hegar, of Germany, and a number of physicians of this country. They are cases of interstitial fibroid tumors, which cases are not amenable to any of the ordinary resources of our art, and which cannot be safely subjected to the usual process for the removal of these tumors. These patients are constantly subject to the danger of death by exhausting hemorrhages; the climacteric follows consequent upon the removal of the ovaries. The tumor gradually shrinks and becomes less. Under these circumstances very little objection can be urged to the operation, as in these cases the patient is usually barren.

There are certain incurable flexions of the uterus in which violent vascular and nervous prostration is produced in the system. Where these flexions are not amenable to any of the other resources of the art, the case being incurable, the extirpation of the ovary is justifiable.

Lastly, allow me to call your attention to a class of cases in which, so far as I know, no one has ever proposed this operation. I had it in my mind eight years ago. It seemed to me then, and it seems to me now, that whenever an obstetrician or gynæcologist feels himself called upon to do the old operation of abdominal section, when there is a contracted pelvis, a rational and proper proceeding under such circumstances would be to ligate and remove the ovaries. It seems to me that during the child-bearing period we have no right to subject a woman who has once gone through the Cæsarean operation to a similar danger under the same circumstances. It would be securing the patient from the possibility of a future necessity of the same operation, and, in my judgment, would be a justifiable proceeding.

*Two Cases of Anterior Displacement of the Ovary, simulating Internal Inguinal Hernia. Battey's Operation.* Dr. G. J. Engelmann, of St. Louis, gave an account of two such cases, a brief abstract of whose remarks is here presented:

In one of these cases to which I allude, the ovary was removed, in the other not, owing to the menopause. In the one the menopause was produced artificially; in the other it was left to nature. The affection as seen by me in these is a rare one. In the one case the disease had existed but a few years; while in the other it was fully developed. In the one mental delusion was but beginning to show itself in the form of melancholia; in the other it had continued for a great many years, and I feared that the nervous system of the patient was hopelessly disturbed. When the ovary was enlarged then it appeared as forming a lump—as the patient expressed it, it had been taken by the physicians to be hernia and a truss was a applied. At no time during the menstrual discharge would this lump appear. I will not enter upon the discussion of the usual lateral displacement, as this has been discussed by Munde, at Baltimore. The much more rare affection of the anterior displacement is only hinted at by him. The first case was a young girl. The ovary was removed and the menopause was brought about by the removal of that one ovary. The patient was twenty four years of age, single, very much emaciated, with a haggard, unsteady look, impaired digestion and mental symptoms tending toward melancholy. She was in moderate fair health until her seventeenth year, when she first menstruated. From the beginning menstruation was scanty, accompanied with severe pain in the side; but the beginning of this very severe mental and physical suffering also dates from a fall from a second story window a few years ago. At the next sickness after the fall she was confined to her bed, and noticed a swelling in the left side. It was excessively tender; she was unable to use the left side. From that time on this lump has confined her to her bed at every menstrual period. She was a girl in circumstances which obliged her to work. Finally she became an inmate of the hospital after passing through the hands of many physicians, regulars, female, homœopathists, everybody. The swelling would also appear after standing, accompanied with much straining. She can wear no light clothing. Bowels usually co-tive, but for from five to six days previous tc the menstrual period she always suffers from diarrhœa. Whenever she wears a truss the pain was very much increased. The right ovary could not be felt; I have never been able to feel it, while the left was distinctly felt, sensitive and slightly enlarged. The lump of which I had heard so much I could not see until during or just before the menstrual flow. Then I saw a slight prominence, which was so exceedingly sensitive that I could not pass my finger over it externally. I found, however, no resistance was to be felt where the ovary is usually felt. I attempted a few times to replace the organs, but it was an attempt without hope of accomplishing the object. After a tonic treatment I removed the offending organ. The ovary was to my mind not enlarged. It was lying in front and to the side of the uterus in the position in which it had been always found before the operation. The patient recovered without a bad symptom.

The second was also a case of anterior displaçement. She was a married lady, forty-six years of age, but also dated her displacement to a fall she had in her twentieth year. She suffered very much in the same way as the former. Her suffering was so intense she was unwilling to continue to live; but the menopause was so near that I urged her to wait. I think I was justified in not removing her ovaries because the menopause was nearly complete.

I judge that the melancholia which existed in both these cases was consequent upon the displacement of the ovaries, as in the case operated on three months ago the melancholia did disappear; in the other, in which melancholia had existed twenty-six years, I suppose neither menopause nor the removal of the ovaries would have removed it.

Dr. Barker, being called upon, said that he felt interested in the subject, and had studied the question closely. I answer the call more for the purpose of eliciting information than from any presumption of communicating it. I have met but three cases of displacement of the ovary in my practice. The first point I wish to know is whether the author of the last paper has any other evidence of the fact that the displacement was produced by the fall than the mere commemorative coincidences he gave. I am inclined to think the displacement was congenital and the fall simply an aggravation. In my three cases I had no fall but believed them to be congenital. One of my cases was a young girl, eighteen years of age, who commenced to menstruate at fourteen and at each menstrual period suffered intensely.

Dr. Wilson—It strikes me that Battey's operation is one of the most heroic operations of the day. It has been abused and will be abused. The selection of proper cases for its use is an important matter. A case came to my attention of a lady who had never menstruated; her uterus was apparently perfect, but with all the means I could bring to bear I never succeeded in getting that young lady to menstruate. She became melancholy and died. Dr. Battey's remarks to-day set me thinking whether if I had removed that lady's ovary the case might not have resulted differently.

Dr. Byford said: I think we have been fortunate in having this question of Battey's operation brought before us by the inventor himself. It is a question which is interesting the profession in all enlightened countries. I was not fortunate enongh to hear Dr. Battey's paper, but it seems to me one of the most profitable views which can be taken of this is the relationship which it bears to the pathology of the general system. I had been acqaint-ed with a patient for two years and knew something about the increase of her tumor. She became hemorrhagic and suffered greatly in health. I finally made a proposition to remove the ovaries. The tumor was large enough to extend above the iliac spine. It had the appearance of being a tumor with only one nucleus of development and situated in the anterior wall of the uterus. After the operation Dr. Trenholme published the case as one that was entirely cured, if I remember right. That patient has been under my care the last year, and her present condition is one almost as miserable as it is possible for a patient to be. Her menstrual hemorrhage is very irregular. It results in consequence of anything like exercise or excitement; she gets so depressed in health as to be at the point of death. The tumor reaches at least two inches above the umbilicus and is about the size it was described to us before the operation. Her condition has been changed in some respects but not improved. The menstrual does not occur, but irregular hemorrhages occur. I believe in the operation however.

Dr. Sims said: In Germany Battey's operatian has been repeatedly performed; in our own country it has advanced slowly; in England it has taken a jump within the past twelve months. Lawson Tait has recently been seized with a mania for the operation, having had twenty-eight cases in eleven months. He reports relief in nearly all the cases, two having died. These two cases, he says, were not well selected. I think Dr. Battey has well classified the cases in which an operation was justifiable. I have recently performed four of Battey's operations. Two of them were having menstrual epileptic affections. Both are doing well. Another was a young lady who had epileptic convulsions at menstruation; she died under the new anæsthetic, bromide of ethyl. A post mortem showed that her death was not the consequence of the operation. Another young lady whom I operated upon was not much benefitted. The lady had had about forty-five to fifty convulsions in a day; she was perfectly rational at these times. Instead of having fifty during the day she now has but half a dozen in the night. My confidence in the operation grows from day to day. I predict for it a grand future when we know how to discriminate the cases in which it is to be employed.

Dr. Wood being requested to speak, said; Having never seen Battey's operation performed, I don't know whether I could tell of any case in which it is properly employed. The author of the paper related a case in which he had removed one ovary and was sorry he did not remove both. I have removed a good many ovaries in my city for various causes but they were always diseased. I have always been glad that I left the healthy ones. For instance, I attended a lady in March, from whom I had removed an ovary in 1867, leaving her the left ovary. From 1867 to 1880 she had borne six living children, three boys and three girls, showing, among other things, that one ovary may produce both sexes. If I had taken out both ovaries that family would have been sacrificed.

Dr. Drysdale remarked that he would be pleased to hear of Dr. Battey as to the statistics of the operation—how often it had proved successful.

Dr. Jackson also requested information as to the effect of stopping the growth of fibrous tumors.

Dr. Battey, in closing the discussion, said: In every instance in which I have removed two ovaries the menopause has occurred. Wherever either of the ovaries was partly left the menses have continued to some extent. We might expect an increased flow of blood to the reproductive organs during menstruation, and thus an increased growth of the tumor might result from that menstrual congestion. It is proposed, theoretically, to stop the growth of the tumor by bringing on the menopause and preventing the patient from being subjected to the menstrual congestion. A consequence of the change of life is the gradual atrophy of the tumor.

In reply to an inquiry, I will say what I call a cure is when we do away with this deplorable perturbation of the nervous and vascular system, which is destroying the life, health and happiness of a patient.—*Cincinnati Lancet and Clinic.*

(TO BE CONCLUDED.)

